# Surface Roughness of CoCr and ZrO_2_ Femoral Heads with Metal Transfer: A Retrieval and Wear Simulator Study

**DOI:** 10.1155/2009/185456

**Published:** 2009-07-01

**Authors:** Alan W. Eberhardt, R. Travis McKee, John M. Cuckler, Donald W. Peterson, Preston R. Beck, Jack E. Lemons

**Affiliations:** ^1^Department of Biomedical Engineering, University of Alabama at Birmingham, Hoehn 370, 1075 13th street S., Birmingham, AL 35294, USA; ^2^Biomet Microfixation, 1520 Tradeport Drive, Jacksonville, FL 32218, USA; ^3^Alabama Spine and Joint Center, 1709 Somerset Circle, Mountain Brook, AL 35213, USA; ^4^Department of Prosthodontics and Dental Biomaterials, University of Alabama at Birmingham, SDB 616, 1919 7th Avenue South, Birmingham, AL 35294, USA

## Abstract

Metal transfer to femoral heads may result from impingement against the metallic acetabular shell following subluxation/dislocation, or when metallic debris enters the articulation zone. Such transfers roughen the head surface, increasing polyethylene wear in total hip replacements. Presently, we examined the surface roughness of retrieved femoral heads with metallic transfer. Profilometry revealed roughness averages in regions of metal transfer averaging 0.380 *μ*m for CoCr and 0.294 *μ*m for ZrO_2_ which were one order of magnitude higher than those from non-implanted controls. Scanning electron microscopy (SEM) revealed adherent transfers on these retrievals, with titanium presence confirmed by electron dispersive spectroscopy. Due to the concern for increased wear, metal transfer was induced on non-implanted heads, which were then articulated against flat polyethylene discs in multidirectional sliding wear tests. Increased polyethylene wear was associated with these specimens as compared to unaltered controls. SEM imaging provided visual evidence that the transfers remained adherent following the wear tests. Pre- and post-test roughness averages exceeded 1 *μ*m for both the CoCr and ZrO_2_ heads. Overall, these results suggest that metal transfer increases the surface roughness of CoCr and ZrO_2_ femoral heads and that the transfers may remain adherent following articulation against polyethylene, leading to increased polyethylene wear.

## 1. Introduction

Metal-on-polyethylene and ceramic-on-polyethylene articulations are common in total hip replacements. The success of these devices depends heavily upon the harder femoral heads remaining smooth over time, since increased roughness of the counterface may dramatically accelerate abrasive polyethylene wear [1–3]. Approximately 3% of total hip arthroplasties are complicated by dislocation within two years of implantation [4], however, which can result in metal transfer and surface roughening when the dislocated or subluxed femoral head contacts the rim of the metallic shell of a modular acetabular component [5, 6]. Entrapment of delaminated porous coating materials [7] or broken prongs [8] within the polyethylene acetabular liner has also been associated with metal transfer in vivo. 

Titanium (Ti) and cobalt-chromium-molybdenum (CoCr) alloys are the primary components in metallic acetabular shells, while porous coatings are often commercially pure titanium or CoCr beads. In either case, Ti or CoCr may transfer to the surface of the harder femoral head material and increase its roughness, leading to increased severe scratching and abrasive wear of polyethylene [9–12]. Müller et al. [13] reported that Ti transfer was abraded away in ceramic-on-ceramic heads; however, for alumina-on-polyethylene it was suggested that the transfer was “more harmful,” retaining its increased roughness and accelerating polyethylene wear. 

The aims of the present study were (1) to study retrieved femoral heads and compare them with nonimplanted controls, and (2) due to the concern for increased wear, to perform an experiment with artificially scratched and nonscratched femoral heads. Surface roughness parameters were quantified in regions of confirmed metal transfer on retrievals for comparison with unused controls, and on intentionally altered femoral heads before and after controlled wear simulations. Scanning electron microscopy and electron dispersive spectroscopy were used to study visual characteristics and the chemical composition of the adherent transfer elements.

## 2. Materials and Methods

### 2.1. Retrievals

**Figure 1:**
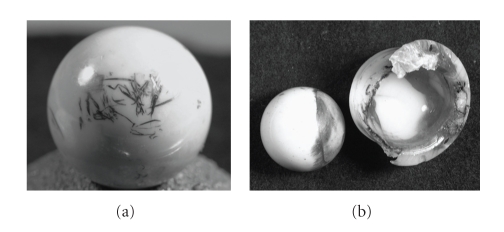
Example retrievals with clear visual evidence of metal transfer on the femoral heads.

Sixteen retrieved femoral heads from four independent manufacturers (Biomet, DePuy, Johnson and Johnson, Richards) were obtained from the University of Alabama Orthopedic Retrieval Laboratory with approval from the UAB Institutional Review Board (IRB no. 01NR, ID no. M-1149). Six CoCr and ten ZrO_2_ heads were selected for visual presence of transfer, a known history of dislocation, wear-through of the polyethylene, or a combination of all three. The extent of damage on the retrievals varied from localized to widespread. Examples of these retrieved components are shown in Figure 1.

This group represented the entire collection of components in our retrieval collection that met the criteria at the time of the study. Available implant and donor data portrayed 28 mm (*n* = 13) and 32 mm (*n* = 3) femoral heads implanted between 0–10 years in patients ranging from 27–75 years old (Table 1). Sterilized specimens were ultrasonically cleaned in acetone to remove any biologic remains and mounted for imaging via a scanning electron microscope with an electron dispersive spectrometer (SEM-EDS, Philips 515, Amsterdam, The Netherlands). The EDS was used to confirm the presence of Ti or other transfer metals on the surface of each femoral head. 

The retrieved specimens were profiled using a Form Talysurf stylus-based contact profilometer (Taylor-Hobson, UK). For each specimen, three 5-mm scans were made at 45-degree increments crossing a randomly selected region of transfer. Each 5-mm scan was then broken down into 1-mm segments, where the surface texture parameters could be determined for isolated regions of transfer. Surface texture parameters including roughness average, *R*_*a*_, root mean square, *R*_*q*_, and skewness, *R*_*sk*_, were calculated according to the equations described in the appendix. The skewness is a measure of symmetry of the amplitude distribution about the mean line, such that a positive *R*_*sk*_ indicates a trend toward peaked surface asperities, while negative *R*_*sk*_ is associated with more troughs and flattened asperities. Eight nonimplanted control heads (4 CoCr, 4 ZrO_2_) were profiled using 5 mm traces without isolating particular regions. Analysis of variance (ANOVA) with two factors (head type: CoCr, ZrO_2_; and treatment: transfer, control) was used to compare the roughness parameters, *R*_*a*_, *R*_*q*_, and *R*_*sk*_, for transfer regions on the retrieved femoral heads and for the unaltered controls.

### 2.2. Induced Titanium Transfer

**Figure 2:**
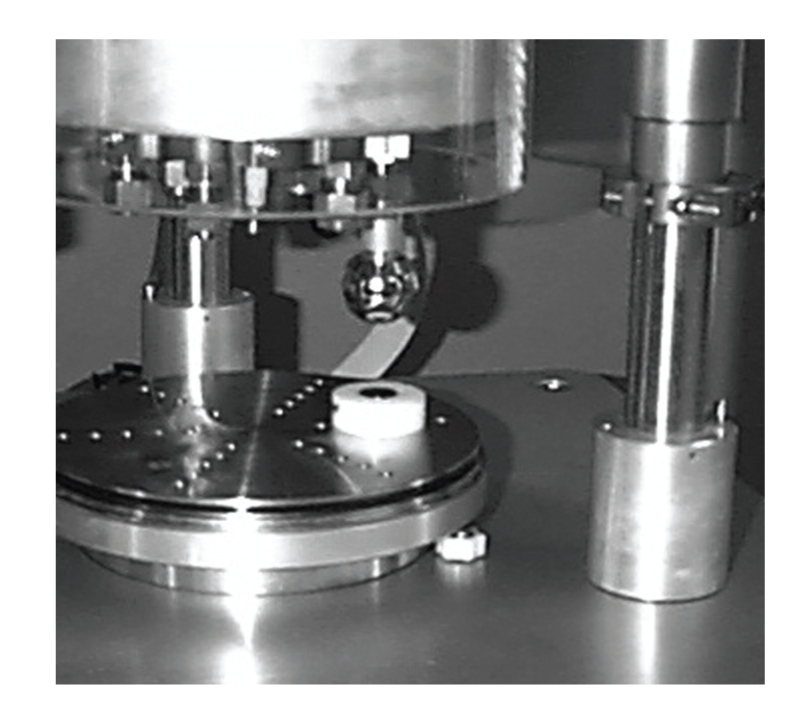
The OrthoPod wear simulator with mounted CoCr test head and transfer disc.

Twelve nonimplanted 32 mm femoral heads were obtained (six CoCr and six ZrO_2_) from three different manufacturers (Biomet, Richards, Zimmer). Six specimens were selected for the induction of transfer using an OrthoPod Friction and Wear Tester (AMTI, Watertown, Mass, USA). Each specimen was fit with a tapered three-centimeter aluminum stem for fixation to the OrthoPod (Figure 2) and programmed to trace a four-centimeter perimeter square pattern on a fixed Ti disc at 100 N for 10 cycles at 0.2 Hertz. Following this articulation, visual confirmation of multidirectional transfer was noted on all six samples. After ultrasonication in an acetone bath, the six femoral heads were imaged with SEM. Surface profilometry was performed using three 5 mm scans at 45 degree increments across the transfers. In this case 2 mm regions were isolated to determine the surface parameters of the transfer. The surface profiles were cataloged as “Pretest.”

Medical grade ultrahigh molecular weight polyethylene (UHMWPE) discs (Piedmont Plastics, Birmingham, Ala, USA), which were machined from ram-extruded bar stock and gamma sterilized (25 kGy) in air served as the counterface material. The selected material represents a uniform grade of UHMWPE that was the standard in the 1980s and 1990s, which is the time period from whence the retrievals were collected. The discs were soaked in bovine serum solution for at least 24 hours prior to testing in order to minimize fluid absorption. Each disc was five centimeters in diameter and 15 millimeters thick and secured to the base plate (disc portion) of the OrthoPod. Wear tests were then performed in which four specimens were tested simultaneously: two control heads with no transfer (one ZrO_2_, one CoCr) and two test heads with induced Ti transfer (one ZrO_2_, one CoCr), along with one unloaded soak-control disc. All tests were performed in a 30% bovine serum solution, with 0.3% sodium azide and 20 mM EDTA added as antibacterial and decalcifying agents, respectively [14]. The solution and test components were kept at 37 ± 2°C with an external circulation heater (Neslab Instruments, Portsmouth NH, USA) for the duration of all tests. 

The articulating femoral heads were programmed to trace a figure-eight pattern onto the surface of the respective polyethylene discs (approximately 50 mm sliding distance/cycle) for 100 000 cycles at 200 N at a rate of 1 Hz. Traditional Hertz equations predicted mean stresses of approximately 25 MPa. Prior to and after each test, the polyethylene discs were weighed with a Mettler Toledo AG245 microbalance (Columbus, Ohio, USA) to the nearest ten-thousandths of a gram. Subtracting the mean control disc weight gain from the difference in individual test disc weights, as indicated in ASTM F-732 [15], the wear loss of each polyethylene specimen was calculated and volumetric wear rates determined. Volumetric wear was derived as *V*_*n*_ = *W*_*n*_/*ρ*, where *W*_*n*_ represents the net weight loss calculated as

(1) $$W_{n}=\left(W_{1}-W_{2}\right)+\left(S_{2}-S_{1}\right),$$

where *W*_1_, *W*_2_, *S*_1_, and *S*_2_ were the pretest, posttest, pretest control, and posttest control polyethylene weights, respectively. The bulk density of the polyethylene was provided by the manufacturer as *ρ* = 0.930 g/cm^3^. 

After wear testing, surface profilometry was performed using three 5 mm scans at 45 degree increments across the transfer region. Again, 2 mm regions were isolated to quantify surface parameters of the transfer. The surface profiles were cataloged as “Posttest.” The roughness parameters and the volumetric wear were compared (pre- and posttest) to investigate changes associated with the articulation of the induced transfer against polyethylene.

## 3. Results

### 3.1. Retrievals

**Figure 3:**
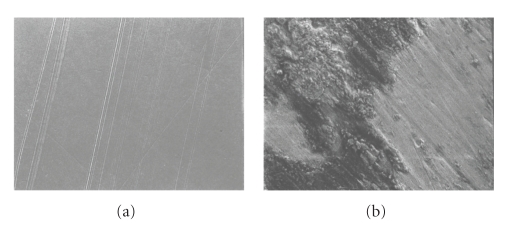
(a) Control CoCr surface reveals residual polishing marks; (b) region of metal transfer (1000X).

**Figure 4:**
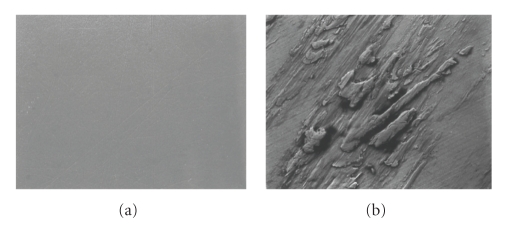
(a) Control ZrO_2_ surfaces appeared smooth; (b) region of metal transfer (1000X).

All the heads tested positive for titanium transfer upon EDS evaluation. The SEM images showed gross topographical alterations of the retrieved femoral heads due to the presence of transfer, as compared to undamaged control surfaces (Figures 3 and 4). Residual polishing scratches were visible running underneath the adherent transfers and occasional polyethylene fragments were found lodged in the transfer surface. Means and standard deviations were determined for roughness parameters, *R*_*a*_, *R*_*q*_, and *R*_*sk*_, associated with the transfer regions on the retrieved femoral heads, and for nonimplanted controls. Roughness averages for the control surfaces were similar at *R*_*a*_ = 0.012–0.013 *μ*m (Table 2). Mean *R*_*a*_ values for the transfers were an order of magnitude greater than the control values (*P* = .01), however, with *R*_*a*_ = 0.38 *μ*m on CoCr and *R*_*a*_ = 0.294 *μ*m on ZrO_2_, *R*_*a*_ was 29% higher on average for the transfer regions on CoCr as compared to ZrO_2_(*P* = .86). Similar trends were observed for *R*_*q*_, with lower average values for controls (*R*_*q*_ = 0.017 *μ*m) as compared to transfer regions (*P* = .01, Table 2). *R*_*q*_ was 49% higher for transfer on CoCr (*R*_*q*_ = 0.54 *μ*m) as compared to ZrO_2_ (*R*_*q*_ = 0.363 *μ*m, *P* = .09). *R*_*sk*_ values were negative for control heads and positive for the transfer specimens (*P* = .01, Table 2). On average, *R*_*sk*_ was 2.34 times greater for transfer regions on the ZrO_2 _ heads than for transfers on the CoCr heads (*P* = .08). 

### 3.2. Induced Transfer

Pretest profilometry indicated that the average roughness values, *R*_*a*_, were an order of magnitude greater for regions of induced transfer (*R*_*a*_ = 1.21 *μ*m for CoCr; *R*_*a*_ = 1.02 *μ*m for ZrO_2_), as compared to the transfer regions on the retrieved devices (Table 3) and two orders of magnitude greater than the surfaces of nonimplanted controls (recall Table 2). A similar trend was observed for the root mean square roughness of the transfers, with *R*_*q*_ = 2.21 *μ*m for CoCr; *R*_*q*_ = 1.69 *μ*m for ZrO_2_. Skewness values were strongly positive for the transfers, with *R*_*sk*_ averaging 3.5 and 3.4 for CoCr and ZrO_2_, respectively.

**Figure 5:**
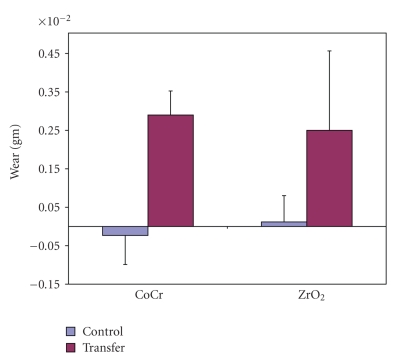
Wear (mass loss) for the control femoral heads and those with induced metal transfer.

Femoral heads with induced transfer were associated with more polyethylene wear than unaltered controls. Polyethylene wear of 2.9 mg and 2.5 mg was measured, respectively, for CoCr and ZrO_2_ heads subjected to articulation against polyethylene for 100 000 cycles (Figure 5). These values were significantly higher (*P* < .003) as compared to those obtained for the controls. Wear associated with the unaltered CoCr and ZrO_2_ specimens averaged −0.23 mg and 0.12 mg, respectively, however, these differences were not statistically significant (*P* > .05). The negative net losses associated with the unaltered CoCr heads indicated that their respective polyethylene discs gained weight during the simulation, which was attributed to fluid absorption during testing. 

**Figure 6:**
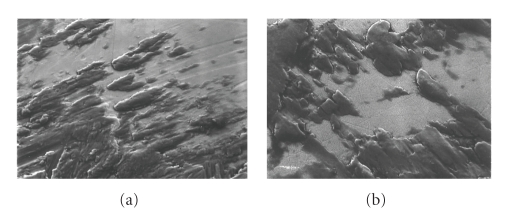
1000X images of postwear transfer regions: (a) CoCr (b) ZrO_2_.

Post-test SEM images of the CoCr and ZrO_2_ samples subjected to the present wear simulation provided visual evidence of adherent transferred metal that closely resembled that of the zirconia retrievals (Figure 6). Mean roughness measures, *R*_*a*_, *R*_*q*_, and *R*_*sk*_ decreased in magnitude following wear testing for all three CoCr heads; however, *R*_*a*_ and *R*_*q*_ actually increased for the ZrO_2_ heads (Table 3). 

## 4. Discussion

The results of this study demonstrated that metal transfer increased the surface roughness above standard manufacturing parameters as seen in nonimplanted CoCr and ZrO_2_ femoral heads. SEM evaluation of retrieved cobalt-alloy and zirconia specimens with EDS-confirmed transfer demonstrated highly altered surface topographies. The average *R*_*a*_ values for the present retrieved devices of around 300 nm compare reasonably with the 181 nm roughness averages reported by Kim et al. [12] for “severely smeared” regions. The negative values of *R*_*sk*_ measured for the control heads were expected for polished surfaces, which are typically extremely flat with residual polishing grooves, that is, valleys. The strongly positive *R*_*sk*_ values for transfer regions reflected a change in surface topography towards an increased percentage of peaking asperities, which would produce a more abrasive surface than the originally finished component. One limitation of the present retrieval analysis is that there was no accurate way to calculate the amount of time the transfers underwent articulation. 

 Luchetti et al. [9] were one of the first to report transfer of metallic debris to a zirconia femoral head from contact with an acetabular shell following hip dislocation. Others have confirmed that Ti or CoCr transfer increases the surface roughness of femoral heads [10], leading to increased scratching and abrasive wear of polyethylene [11, 12]. How well the transfer adheres to the femoral head and maintains its roughness during subsequent articulation is likely to affect the long-term wear rates and may depend upon the femoral head material. While Ti transfer may abrade away in ceramic-on-ceramic heads, such transfer retains its increased roughness in ceramic-on-polyethylene devices accelerating polyethylene wear [13]. While Schuh et al. [10] reported no adherent Ti on a scratched CoCr retrieval, the present results suggested that titanium may remain adherent to CoCr and zirconia surfaces in contact with polyethylene counterfaces, thereby increasing surface roughness and potentially increasing polyethylene wear.

The present in vitro wear simulation demonstrated more polyethylene wear with femoral surfaces roughened by metal transfer than with control surfaces, consistent with general conclusions made for lubricated wear couples [1, 2]. These results demonstrated significant contributions of the transfer element to wear for both CoCr and ZrO_2_ femoral heads, consistent with those of Kim et al. [12] for alumina heads. Roughness measures for the induced transfer regions were an order of magnitude higher than for transfers found on retrievals, however, indicating more severely roughened surfaces among the induced transfer specimens as compared to the retrievals. This finding indicates a possible limitation of the present in vitro study, which appears to represent a much more severe condition than encountered in-vivo. Another limitation of the present study is that the femoral head-on-disk wear test that was employed is, at best, a test that demonstrates the potential for increased UHMWPE wear when a CoCr or zirconia counterface is artificially modified with metal transfer. The tests were conducted for an extremely limited number of cycles (100 000) and did not accurately replicate hip joint conditions; therefore, these results should be considered as a preliminary step to in vitro hip wear simulator tests.

The present postwear specimens displayed adherent transfers. Lowered *R*_*sk*_ values demonstrated some reduction in peak heights for regions of transfer after wear simulation; however, *R*_*a*_ and *R*_*q*_ were nearly unchanged suggesting that the transfer elements remained rough with only a slightly less irregular (sharp) morphology. Thus the surface profiles for tested pieces would still be considered abrasive by clinical standards. It is difficult to estimate the clinical significance of such femoral head damage; however, greater polyethylene wear can be expected to increase the occurrence of osteolysis [16–18]. Clinically, the overall results of the present study suggest that CoCr and zirconia femoral heads that have experienced dislocation or subluxation should be considered suboptimal wear surfaces and should be exchanged for new femoral heads if revision is necessary. Because metal transfer may not be as visibly apparent on CoCr heads, clinicians should take extra care to avoid impingement of these devices against the metallic backing during operative procedures. Surgeons treating patients with recurrent subluxation or dislocation should closely monitor the patients with regular X-rays in order to detect and intervene if accelerated wear or osteolysis should appear.

## Figures and Tables

**Table 1:** Retrieval information (blank cells indicate unavailable information).

Implant data			Donor data			
Manufacturer	Size (mm)/Material	Mos. in vivo	Age (yrs)	Sex	Weight (lbs.)	Height (inches)
J&J	28/CoCr	36	55	M	145	70
Richards	28/CoCr	60	75	F	119	62
Richards	28/CoCr	30	49	F	215	65
Depuy	32/CoCr	108	82	F	145	65
Biomet	28/CoCr	0.3	60	F		
	28/CoCr			M	143	69

Richards	28/ZrO_2_	54				
Richards	28/ZrO_2_	48	39	M	215	72
Biomet	28/ZrO_2_	12	27			
Richards	28/ZrO_2_	60	68	M		
Biomet	28/ZrO_2_	2	46	M	139	69
Richards	28/ZrO_2_			F		
Richards	32/ZrO_2_	120	40	F		
Biomet	28/ZrO_2_					
Biomet	28/ZrO_2_		49	M	185	73
	32/ZrO_2_		50	M		

**Table 2:** Surface parameters for retrievals (mean ± standard deviation).

Head type	*R*_*a*_ (*μ*m)	*R*_*q*_ (*μ*m)	*R*_*sk*_
CoCr control	0.012 ± 0.002	0.016 ± 0.004	−0.85 ± 1.09
ZrO_2_ control	0.013 ± 0.002	0.018 ± 0.003	−0.204 ± 0.114
CoCr transfer	0.380 ± 0.308	0.540 ± 0.512	0.597 ± 1.319
ZrO_2_ transfer	0.294 ± 0.294	0.363 ± 0.387	1.397 ± 1.365

**Table 3:** Pre- and posttest surface parameters for induced-transfer specimens and controls used in wear tests.

Material	Pretest			Posttest		
	*R*_*a*_ (*μ*m)	*R*_*q*_ (*μ*m)	*R*_*sk*_	*R*_*a*_ (*μ*m)	*R*_*q*_ (*μ*m)	*R*_*sk*_
CoCr	1.213 ± .156	2.208 ± .276	3.500 ± .638	1.010 ± .180	1.630 ± .250	2.800 ± .231
ZrO_2_	1.024 ± .295	1.694 ± .504	3.400 ± .505	1.327 ± .960	2.034 ± 1.381	2.767 ± .437

## Appendix

### Roughness Measures

The roughness average, *R*_*a*_, represents a universally recognized parameter of roughness. It is the arithmetic mean of the vertical departures (both above and below) from the centerline of the segment of profile under examination [19] and is determined by integrating the profile function and dividing by the length of the scan:

(A.1) $$R_{a}=\frac{1}{L}\int \left|y\left(x\right)\right|dx,$$

where *L* is the length of the scan and *y* is the vertical displacement from the centerline as a function of position, *x*. The root mean square parameter, *R*_*q*_, is defined as the square root of the mean of the squares of the *R*_*a*_ values for a particular scan. It is derived from the scan mathematically in similar fashion,

(A.2) $$R_{q}=\sqrt{\frac{1}{L}\int \;y{\left(x\right)}^{2}dx.}$$

While these are the most widely utilized parameters for surface roughness, they do not recognize valleys from peaks over the course of the scan. In order to more accurately approximate the surface roughness as a function of peak height and positive deviations from the centerline, *R*_*sk*_ parameters were tabulated for each scan. *R*_*sk*_ represents the skewness of the profile and is a measure of symmetry of the amplitude distribution about the mean line, or

(A.3) $$R_{sk}=\frac{1}{nR_{q}^{3}}\sum {\left(y_{i}-Y\right)}^{3},$$

where *n* serves as the number of coordinate values made (*y* values) and *Y* is the numerical value of the mean line. A positive skewness measure denotes asymmetry above the mean line representing a greater number of peaking asperities. Negative skewness values represent asymmetry below the mean line, or greater troughs in the profile.
